# Cincho-Quinine

**Published:** 1874-12

**Authors:** 


*Cinquo-Quinine.*

This new antiperiodic, prepared by Billings, Clapp & Co., Boston, seems to be rapidly gaining the confidence of the profession. We think this new agent should be thoroughly tested by the profession. It claims to be equal to the sulphate of quinine, and in some cases superior. It is obtained from Peruvian bark, and contains all the alkaloids of the bark. It is put up by a well-known and highly respectable house, and we believe will prove an invaluable addition to our list of therapeutical agents. Remember that Billings, Clapp & Co. is one of the largest and most reliable drug houses in Boston.

